# External Validation of Surrogate Indices of Fatty Liver in the General Population: The Bagnacavallo Study

**DOI:** 10.3390/jcm10030520

**Published:** 2021-02-01

**Authors:** Francesco Giuseppe Foschi, Fabio Conti, Marco Domenicali, Pierluigi Giacomoni, Alberto Borghi, Vittoria Bevilacqua, Lucia Napoli, Dante Berardinelli, Mattia Altini, Alessandro Cucchetti, Giorgio Ercolani, Andrea Casadei-Gardini, Stefano Bellentani, Amalia Gastaldelli, Claudio Tiribelli, Giorgio Bedogni, Bagnacavallo Study Group

**Affiliations:** 1 Azienda Unità Sanitaria della Romagna, Ospedale degli Infermi, 48018 Faenza, Italy; francesco.foschi@auslromagna.it (F.G.F.); fabio.conti@auslromagna.it (F.C.); pierluigi.giacomoni@alice.it (P.G.); vittoria.bevilacqua@auslromagna.it (V.B.); lucia.napoli@auslromagna.it (L.N.); dante.berardinelli@auslromagna.it (D.B.); mattia.altini@auslromagna.it (M.A.); 2 Faculty of Medicine and Surgery, University of Bologna, 40138 Bologna, Italy; marco.domenicali@gmail.com (M.D.); alberto.borghi@auslromagna.it (A.B.); alessandro.cucchett2@unibo.it (A.C.); giorgio.ercolani@auslromagna.it (G.E.); 3 Faculty of Medicine and Surgery, University of Modena and Reggio Emilia, 41121 Modena, Italy; casadeigardini@gmail.com; 4 Italian Liver Foundation, 34012 Basovizza, Italy; bellentanistefano@gmail.com (S.B.); ctliver@fegato.it (C.T.); 5 Italian National Research Council, 56124 Pisa, Italy; amalia@ifc.cnr.it

**Keywords:** cross-sectional study, diagnostic techniques and procedures, validation study, fatty liver, non-alcoholic fatty liver disease

## Abstract

We externally validated the fatty liver index (FLI), the lipid accumulation product (LAP), the hepatic steatosis index (HSI), and the Zhejiang University index (ZJU) for the diagnosis of fatty liver (FL) and non-alcoholic fatty liver disease (NAFLD) in the general population. The validation was performed on 2159 citizens of the town of Bagnacavallo (Ravenna, Italy). Calibration was evaluated by calculating the calibration slope and intercept and by inspecting calibration plots; discrimination was evaluated using the c-statistic. The average calibration slope was 1 and the average intercept was 0 for all combinations of outcomes and indices. For the diagnosis of FL, the c-statistic was 0.85 for FLI, 0.83 for ZJU, 0.82 for HSI, and 0.80 for LAP; for the diagnosis of NAFLD, the c-statistic was 0.77 for FLI, 0.76 for ZJU, 0.75 for HSI, and 0.74 for LAP. All indices were strongly correlated with each other. In conclusion, FLI, LAP, HSI, and ZJU perform similarly well to diagnose FL and NAFLD in the Bagnacavallo population, even if FLI has a small advantage as discrimination is concerned.

## 1. Introduction

Fatty liver (FL), the most common liver disease worldwide, has been classified into non-alcoholic fatty liver disease (NAFLD) and alcoholic fatty liver disease (AFLD) for almost 40 years [1]. Such dichotomization has been increasingly criticized, so an international panel of experts has recently proposed to abandon the NAFLD definition, adopting instead the more comprehensive definition of metabolic dysfunction-associated fatty liver disease (MAFLD), which has the advantage of being independent of alcohol intake [2,3,4].

Independently of its etiology, FL is operationally defined as visible steatosis in more than 5% of hepatocytes at liver biopsy or as an intrahepatic triglyceride content of at least 5.6% at magnetic resonance spectroscopy (MRS) or magnetic resonance imaging [5]. Liver biopsy can be performed only in selected patients followed at secondary or tertiary care centers and the use of magnetic resonance techniques is restricted to a few research centers because of its cost [5].

The most common method to diagnose FL in clinical practice and epidemiological research is liver ultrasonography (LUS) [5]. Another method is the measurement of the controlled attenuation parameter using FibroScan, which is however expensive and not readily available [5]. When LUS is not available, the presently suggested method to diagnose FL is the calculation of surrogate indices of FL [5]. Such indices are referred to as “biomarkers” by the European Association for the Study of the Liver (EASL) guidelines on NAFLD [5].

As for any diagnostic test, the performance of FL biomarkers should be externally validated in terms of calibration and discrimination [6,7,8]. However, as it happens for most diagnostic research [6,7,8], calibration is often neglected by the available validation studies of FL biomarkers, with some notable exceptions [9,10,11]. Calibration is nonetheless the primary requirement to perform decision-making and inform patients, and a test with high discrimination but low (or unknown) calibration is not clinically useful [6,7,8].

Using LUS as the reference method, we evaluated the calibration and discrimination of FL biomarkers at diagnosing FL and NAFLD in the general population of the Bagnacavallo study [12,13,14]. The study was reported following the TRIPOD (Transparent reporting of a multivariable prediction model for individual prognosis or diagnosis) guidelines [6] (Appendix A).

## 2. Subjects and Methods

### 2.1. Source of Data

The validation of FL biomarkers was performed using data collected during the Bagnacavallo Study [12,13,14]. The Bagnacavallo study was aimed at evaluating the prevalence of and the risk factors for FL in a cross-section of the general population of a Northern Italy town, and at developing a cohort of subjects from the general population where the association between FL and incident health outcomes could be studied. The study was approved by the Ethics Committee of Area Vasta Romagna-IRST (reference number 112), and all subjects gave their written informed consent.

### 2.2. Participants

As described in detail elsewhere [13], 3933 citizens of the town of Bagnacavallo (Ravenna, Italy) aged 30 to 60 years, were studied between October 2005 and March 2009. Altered liver enzymes were defined as alanine transaminase (ALT) > 40 U/L and/or aspartate transaminase (AST) > 37 U/L, i.e., the upper limit of normal of the laboratory. After exclusion of subjects with hepatitis B virus (HBV) infection, hepatitis C virus (HCV) infection, and unavailability of LUS, the Bagnacavallo cross-sectional analysis was performed on 349 citizens with and 1810 without altered liver enzymes [13]. The same sample of 2159 citizens was analyzed here. All participants underwent a detailed clinical history and physical examination [15]. Alcohol intake was assessed by interview [13]. Weight and height were measured following international guidelines [16] and waist circumference was measured at the midpoint between the last rib and the iliac crest [17]. Body mass index (BMI) was calculated as weight (m)/height (m)^2^ [18]. Performed blood tests included: (1) glucose; (2) triglycerides; (3) total cholesterol; (4) high-density lipoprotein (HDL) cholesterol; (5) low-density lipoprotein (LDL) cholesterol; (6) ALT; (7) AST; (8) GGT. Systolic and diastolic blood pressure was measured using a sphygmomanometer following international guidelines. (The recommended method of measurement of blood pressure remained the same during the study period.) The metabolic syndrome was diagnosed using the harmonized international definition [19].

### 2.3. Outcomes

The primary outcome was LUS-diagnosed FL and the secondary outcome was LUS-diagnosed NAFLD. We focused on LUS not because it is the gold-standard method but because it is the method most commonly employed [5]. We did not consider other diagnostic methods besides LUS, e.g., MRS, because we wanted to control the error attributable to the choice of the reference method.

LUS was performed by five experienced physicians using the same methodology employed by the Dionysos Nutrition & Liver Study [15]. In detail, normal liver was defined as the absence of liver steatosis or other liver abnormalities; light FL was defined as the presence of slight bright liver or hepatorenal echo contrast without intrahepatic vessels blurring and no deep attenuation; moderate FL as the presence of mild bright liver or hepatorenal echo contrast without intrahepatic vessel blurring and with deep attenuation; and severe FL as diffusely severe bright liver or hepatorenal echo contrast, with intrahepatic vessels blurring (no visible borders) and deep attenuation without visibility of the diaphragm. For the present analysis, FL was coded as 0 = normal liver and 1 = any degree of FL.

NAFLD was defined as FL associated with ethanol intake <2 alcohol units (20 g/day) in women and <3 alcohol units (30 g/day) in men testing negative for HBV surface antigen and anti-HCV antibodies and not under treatment with steatogenic drugs [5]. Alcoholic fatty liver disease (AFLD) was defined as FL associated with ethanol intake ≥2 alcohol units in women and ≥3 alcohol units in men testing negative for hepatitis B surface antigen and anti-HCV antibodies and not under treatment with steatogenic drugs [5]. For the present analysis, NAFLD was coded as 0 = normal liver or AFLD and 1 = NAFLD.

### 2.4. Predictors

We identified five non-patented FL biomarkers, developed using LUS as the reference method, for potential inclusion into the study: fatty liver index (FLI) [20], lipid accumulation product (LAP) [17], hepatic steatosis index (HSI) [21], Zhejiang University index (ZJU) [22], and the index of non-alcoholic steatohepatitis (ION) [23].

FLI is suggested by the European Association for the Study of the Liver (EASL) as a “biomarker” of liver steatosis [5]. Other “biomarkers” suggested by the EASL are SteatoTest [24], which is based on a proprietary formula and could not be validated here, and the NAFLD-liver fat score (NAFLD-LFS) [25], which was developed using MRS as the reference method and was therefore not considered here. We were also unable to calculate NAFLD-LFS because insulin, which is a required predictor of NAFLD-LFS, was available only in 1415 (66%) of our 2159 subjects. For the same reason and because of the unavailability of hip circumference, we could not to calculate the ION index, which requires both insulin and the waist-to-hip ratio. (We could have imputed the missing values of insulin [12], but we chose not to do so because insulin is a key predictor of FL [20], and key predictors should not be missing when developing or validating prediction models [7].)

FLI and LAP were developed to predict FL while HSI and ZJU were developed to predict NAFLD. All biomarkers were developed in cross-sections of individuals from the general population (FLI, LAP) or health-care facilities (HSI, ZJU), by matching individuals with FL or NAFLD to individuals without it. The formulae for calculating FLI, LAP, HSI and ZJU are given in Table A3 of Appendix B.

### 2.5. Sample Size

We did not perform any formal sample size calculation but were quite confident that, with 896/2159 (42%) cases of FL and 567/2159 (26%) cases of NAFLD, we could attain a precise assessment of the performance of the biomarkers [13]. At least 200 events and 200 non-events are in fact required for reasonable external validation of model performance [6,7].

### 2.6. Missing Data

There were no missing data.

### 2.7. Statistical Analysis

Most continuous variables were not Gaussian-distributed, and all are reported as median (50th percentile) and interquartile range (25th and 75th percentiles). Discrete variables are reported as the number and proportion of subjects with the characteristic of interest.

Calibration was evaluated by applying Van Calster’s three-level hierarchy [8,26]. Level 1 of this hierarchy is “mean calibration” or “calibration-in-the-large”, which compares the observed event rate with the average predicted risk; level 2 is “weak calibration”, which consists of a logistic calibration analysis testing whether the calibration slope is 1 and the calibration intercept is 0, and is aimed at detecting overestimation or underestimation of risk; lastly, level 3 is “moderate calibration”, which uses a “calibration plot” to test whether the predicted risks correspond to the observed event rates. Such a graph plots the predicted (expected) outcome probabilities (x-axis) against the observed outcome frequencies (y-axis). As suggested by the TRIPOD guidelines, we performed the calibration using tenths of the predicted risk and superimposed a line obtained by locally weighted scatterplot smoothing [6]. A well-calibrated model shows predictions lying or around the 45° line of the calibration plot [6]. Discrimination was evaluated using Harrell’s c-statistic [27].

Statistical analysis was performed using Stata 16.1 (Stata Corporation, College Station, TX, USA) with the *pmcalplot* module [28], and R 4.0.3 (R Core Team 2020, Vienna, Austria) with the *val.prob.ci.2* function [8]. R code was run from within Stata using the *rcall* package [29].

## 3. Results

### 3.1. Study Population

The measurements of the 2159 citizens who took part in the study are given in Table 1 and are discussed in greater detail elsewhere [12]. FL was diagnosed in 896/2159 (42%, 95% CI 39 to 44%) and NAFLD in 567/2159 (26%, 24 to 28%) citizens.

### 3.2. Diagnosis of FL

The average expected rate of FL (42%) equaled the average observed rate (42%) for all biomarkers, showing a satisfactory mean calibration (Table 2).

**Figure 1:**
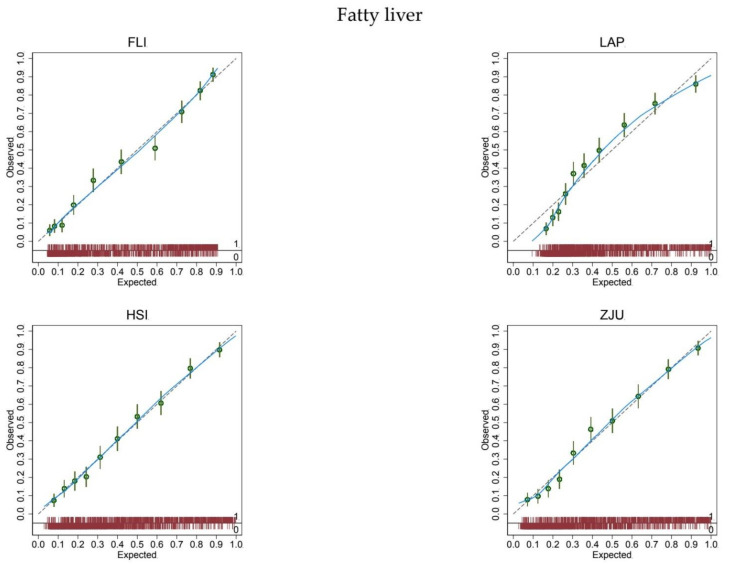
Calibration plots for the diagnosis of fatty liver.

At logistic calibration, the average calibration slope was 1 and the average intercept was 0 for all biomarkers, showing a satisfactory weak calibration (Table 2). Lastly, the examination of calibration plots (Figure 1) showed an acceptable profile of moderate calibration for all predictors. FLI had the highest (0.85) c-statistic, followed by ZJU (0.83), HSI (0.82), and LAP (0.80).

The expected (predicted) risk is divided into 10 equally sized groups (tenths). The green dots and spikes on the diagonal line are average risks and 95% confidence intervals. The dotted line is the reference line of calibration. The blue line connecting the green dots is obtained by locally weighted scatterplot smoothing. The red spike plot at the bottom gives the distribution of fatty liver (0 = no; 1 = yes). Abbreviations: FLI = fatty liver index; LAP = lipid accumulation product; HSI = hepatic steatosis index; ZJU = Zhejiang University index.

### 3.3. Diagnosis of NAFLD

**Figure 2:**
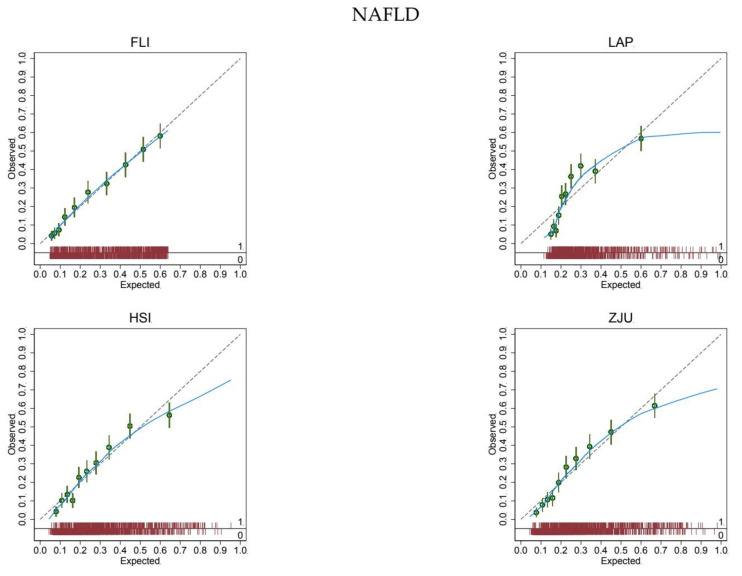
Calibration plots for the diagnosis of non-alcoholic fatty liver disease.

The expected rate of NAFLD (26%) equaled the observed rate (26%) for all biomarkers, showing a satisfactory mean calibration (Table 2). At logistic calibration, the average calibration slope was 1 and the average intercept was 0 for all biomarkers, showing a satisfactory weak calibration (Table 2). Lastly, the examination of calibration plots showed an acceptable profile of moderate calibration for all predictors (Figure 2). FLI had the highest (0.77) c-statistic, followed by ZJU (0.76), HSI (0.75), and LAP (0.74).

The expected (predicted) risk is divided into 10 equally sized groups (tenths). The green dots and spikes on the diagonal line are average risks and 95% confidence intervals. The dotted line is the reference line of calibration. The blue line connecting the green dots is obtained by locally weighted scatterplot smoothing. The red spike plot at the bottom gives the distribution of NAFLD (0 = no; 1 = yes). Abbreviations: NAFLD = non-alcoholic fatty liver disease; FLI = fatty liver index; LAP = lipid accumulation product; HSI = hepatic steatosis index; ZJU = Zhejiang University index.

### 3.4. Association between Biomarkers

**Figure 3:**
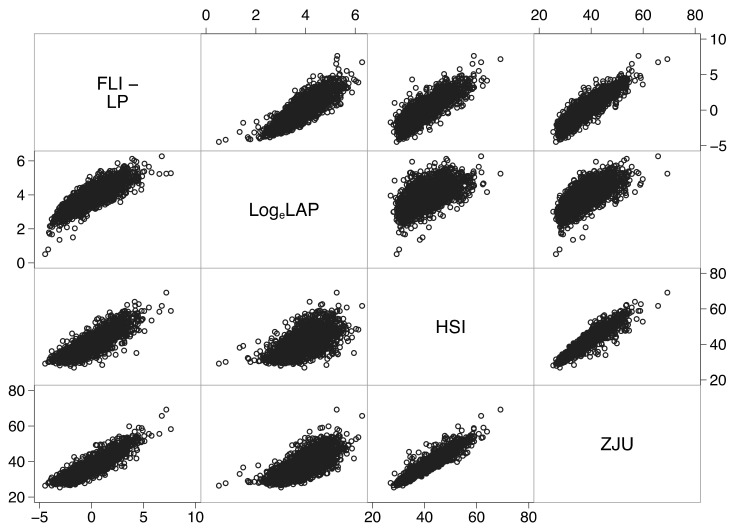
Correlation matrix showing strong associations between all biomarkers. Abbreviations: FLI-LP = fatty liver index-linear predictor (see Table A3 of Appendix B); Log_e_LAP = natural logarithm of the lipid accumulation product; HSI = hepatic steatosis index; ZJU = Zhejiang index.

Further analysis revealed a strong association between all biomarkers (Figure 3), partially explained by the use of the same or highly correlated predictors (Table A2 of Appendix B).

For instance, the linear predictor of FLI explained 72% of the variance of HSI, 81% of the variance of ZJU, and 51% of the variance of log_e_-transformed LAP. Moreover, ZJU explained 89% of the variance of HSI. The similar performance of these biomarkers at diagnosing FL and NAFLD (Table 1) is thus likely to be partially explained by their underlying mutual association.

## 4. Discussion

In the present study, we took advantage of the Bagnacavallo cross-sectional study of liver disease [13] to externally validate FLI [20], LAP [17], HSI [21], and ZJU [22] for the diagnosis of FL and NAFLD in the general population. All biomarkers showed an acceptable mean, weak, and moderate calibration for the diagnosis of FL (Figure 1 and Table 2) and NAFLD (Figure 2 and Table 2) [8].

We hypothesized that FLI would perform better than LAP, HSI, and ZJU at diagnosing FL and possibly NAFLD in the present population. (We had some reservations about NAFLD because FLI was purposely developed to predict FL.) Our hypothesis was based on the fact that FLI was developed in the general population of a town (Campogalliano, Modena, Italy) similar to the one studied here (Bagnacavallo, Ravenna, Italy) [20]. We expected, however, in line with the available evidence [9,10,11], that FLI had to be recalibrated for proper use in the Bagnacavallo population. We were thus surprised to find that FLI had a satisfactory mean, weak and moderate calibration, and that it could be applied without modification to the Bagnacavallo population for the diagnosis of both FL and NAFLD. We were even more surprised to find that biomarkers (LAP, HSI and ZJU) developed in different populations (US, Korea and China) showed a satisfactory profile of mean, weak, and moderate calibration in the Bagnacavallo population.

The strengths of the present study are that it was performed in a representative sample of the general population, that it enrolled a high number of subjects, and that it had a high observed event rate for both FL and NAFLD. A sample size of at least 200 subjects with and 200 without the outcome of interest is presently suggested for proper validation of a diagnostic test [7]. With its 896 citizens with and 1263 without FL, and 567 citizens with and 1592 without NAFLD, the Bagnacavallo Study is thus in an excellent position to serve as a platform to externally validate biomarkers of FL.

A limitation of the present study is the unavailability of some predictors needed to calculate the ION index [23], which was one of the biomarkers that we identified as theoretically suitable for validation in this population. The ION index employs insulin, which was available only in a subsample of subjects, and hip circumference, which was not measured in the Bagnacavallo study. Moreover, the partial availability of insulin and the unavailability of C-reactive protein impeded us to diagnose MAFLD and to evaluate the performance of FL biomarkers at diagnosing this newly proposed entity, which is expected to attract much attention in coming years [2,3]. Furthermore, even if we chose to include studies that used only LUS to diagnose FL to reduce the error attributable to the choice of the reference method, LUS is known to offer an accurate assessment of FL only starting from an intrahepatic triglyceride content of 10% [30].

External calibration is more important than discrimination at establishing the value of a test for a given diagnosis, but has not been taken into account by most diagnostic studies of FL biomarkers, with some notable exceptions [9,10,11]. This is not to say that discrimination is irrelevant because, in the presence of an acceptable calibration, the greater discrimination is preferable. Another problem of most diagnostic studies is that they compare an externally derived predictor with an internally derived one and go on to declare the latter superior to the former [6]. This is, however, largely expected on both theoretical and empirical grounds, and is one of the primary reasons why external validation of diagnostic models is so important [6,7,8].

The similar performance of the biomarkers at diagnosing FL and NAFLD in the present study is likely to be partially explained by their underlying mutual association (Figure 3). This finding, which awaits replication in other populations, suggests that the same set of predictors may be employed to develop a common algorithm for the prediction of FL. The re-estimation of some or all regression coefficients or the updating of the algorithm with new predictors should be done only if it increases its performance [7].

## 5. Conclusions

In conclusion, FLI, LAP, ZJU, and HSI show similar performance at diagnosing FL and NAFLD in the Bagnacavallo population, even if FLI has greater discrimination. FLI, LAP, ZJU, and HSI are strongly associated, which is likely to explain their similar performance. Further studies are needed to evaluate the use of these surrogate indices for the diagnosis of MAFLD [31], the diagnostic entity which is expected to attract much attention in coming years [2,3,4]. We hope that the findings of the present study will stimulate the routine reporting of calibration in future studies of surrogate indices of fatty liver because it is the use of this metric that made it possible to detect their similar performance in the Bagnacavallo population.

## Figures and Tables

**Table 1:** Measurements of the study subjects.

	*n* = 2159
Altered liver enzymes	349 (16.2%)
Male sex	1079 (50.0%)
Age (years)	49 (41; 56)
Body mass index (kg/m^2^)	25.5 (23.0; 29.0)
Normal liver	1263 (58.5%)
Fatty liver	896 (41.5%)
Non-alcoholic fatty liver disease	567 (26.3%)
Alcoholic fatty liver disease	329 (15.2%)
Waist circumference (cm)	101.0 (94.0; 108.0)
Glucose (mg/dL)	89 (83; 97)
Triglycerides (mg/dL)	102 (71; 153)
Total cholesterol (mg/dL)	209 (185; 235)
HDL-cholesterol (mg/dL)	59 (49; 71)
LDL-cholesterol (mg/dL)	128 (105; 152)
Systolic blood pressure (mm Hg)	130 (120; 140)
Diastolic blood pressure (mm Hg)	80 (80; 90)
Metabolic syndrome	615 (28.5%)
Alanine transaminase (U/L)	22 (16; 32)
Aspartate transaminase (U/L)	22 (18; 26)
Gamma-glutamyltransferase (U/L)	19 (13; 32)
Alcohol intake (units/day)	2 (0; 4)
Fatty liver index (FLI)	46 (21; 76)
Lipid accumulation product (LAP)	44 (28; 75)
Hepatic steatosis index (HSI)	39 (35; 44)
Zhejiang University index (ZJU)	36 (33; 41)

Continuous variables are reported as median (50th percentile) and interquartile range (25th and 75th percentiles). Discrete variables are reported as the number and proportion of subjects with the characteristic of interest. HDL = high-density lipoprotein; LDL = low-density lipoprotein.

**Table 2:** Calibration and discrimination of the fatty liver index, lipid accumulation product, hepatic steatosis index and Zhejiang University index at diagnosing fatty liver and non-alcoholic fatty liver disease.

	**Fatty Liver**			
	FLI	LAP	HSI	ZJU
Expected event rate ^†^	0.42 (0.40 to 0.43)	0.42 (0.40 to 0.43)	0.42 (0.40 to 0.43)	0.42 (0.40 to 0.43)
Calibration intercept	0.00 (−0.11 to 0.11)	0.00 (−0.10 to 0.10)	0.00 (−0.10 to 0.10)	0.00 (−0.10 to 0.10)
Calibration slope	1.00 (0.92 to 1.08)	1.00 (0.89 to 1.11)	1.00 (0.91 to 1.09)	1.00 (0.91 to 1.09)
C-statistic	0.85 (0.84 to 0.87)	0.80 (0.78 to 0.82)	0.82 (0.80 to 0.83)	0.83 (0.81 to 0.85)
	**Non-Alcoholic Fatty Liver Disease**			
	FLI	LAP	HSI	ZJU
Expected event rate ^††^	0.26 (0.25 to 0.28)	0.26 (0.24 to 0.28)	0.26 (0.25 to 0.28)	0.26 (0.25 to 0.28)
Calibration intercept	0.00 (−0.11 to 0.11)	0.00 (−0.10 to 0.10)	0.00 (−0.10 to 0.10)	0.00 (−0.10 to 0.10)
Calibration slope	1.00 (0.89 to 1.11)	1.00 (0.84 to 1.16)	1.00 (0.88 to 1.12)	1.00 (0.88 to 1.12)
C-statistic	0.77 (0.75 to 0.79)	0.74 (0.71 to 0.76)	0.75 (0.72 to 0.77)	0.76 (0.74 to 0.78)

Values are averages and 95% confidence intervals; ^†^ vs. observed event rate of 0.42 (0.39 to 0.44); ^††^ vs. observed event rate of 0.26 (0.24 to 0.28). Abbreviations: FLI = fatty liver index; LAP = lipid accumulation product; HSI = hepatic steatosis index; ZJU = Zhejiang University index.

## Data Availability

The data presented in this study are available on reasonable request from the corresponding author.

## Appendix A

**Table A1:** TRIPOD (Transparent reporting of a multivariable prediction model for individual prognosis or diagnosis) checklist [6].

Section/Topic	Item	Checklist Item	Page
Title and abstract			
Title	1	Identify the study as developing and/or validating a multivariable prediction model, the target population, and the outcome to be predicted.	1
Abstract	2	Provide a summary of objectives, study design, setting, participants, sample size, predictors, outcome, statistical analysis, results, and conclusions.	1
Introduction			
Background and objectives	3a	Explain the medical context (including whether diagnostic or prognostic) and rationale for developing or validating the multivariable prediction model, including references to existing models.	2
	3b	Specify the objectives, including whether the study describes the development or validation of the model or both.	2
Methods			
Source of data	4a	Describe the study design or source of data (e.g., randomized trial, cohort, or registry data), separately for the development and validation data sets, if applicable.	2
	4b	Specify the key study dates, including start of accrual; end of accrual; and, if applicable, end of follow-up.	2
Participants	5a	Specify key elements of the study setting (e.g., primary care, secondary care, general population) including number and location of centres.	2
	5b	Describe eligibility criteria for participants.	2
	5c	Give details of treatments received, if relevant. Comment: no treatment was administered outside good clinical practice.	NAP
Outcome	6a	Clearly define the outcome that is predicted by the prediction model, including how and when assessed.	3
	6b	Report any actions to blind assessment of the outcome to be predicted. Comment: biomarkers were calculated after the end of the study.	NAP
Predictors	7a	Clearly define all predictors used in developing or validating the multivariable prediction model, including how and when they were measured.	13
	7b	Report any actions to blind assessment of predictors for the outcome and other predictors. Comment: biomarkers were calculated after the end of the study.	NAP
Sample size	8	Explain how the study size was arrived at.	3
Missing data	9	Describe how missing data were handled (e.g., complete-case analysis, single imputation, multiple imputation) with details of any imputation method.	3
Statistical analysis methods	10c	For validation, describe how the predictions were calculated.	13
	10d	Specify all measures used to assess model performance and, if relevant, to compare multiple models.	4
	10e	Describe any model updating (e.g., recalibration) arising from the validation, if done. Comment: there was no need to recalibrate any biomarker.	NAP
Risk groups	11	Provide details on how risk groups were created, if done.	4
Development vs. validation	12	For validation, identify any differences from the development data in setting, eligibility criteria, outcome, and predictors.	3, 12
Results			
Participants	13a	Describe the flow of participants through the study, including the number of participants with and without the outcome and, if applicable, a summary of the follow-up time. A diagram may be helpful.	2
	13b	Describe the characteristics of the participants (basic demographics, clinical features, available predictors), including the number of participants with missing data for predictors and outcome.	2
	13c	For validation, show a comparison with the development data of the distribution of important variables (demographics, predictors and outcome).	2
Model performance	16	Report performance measures (with CIs) for the prediction model.	5
Model-updating	17	If done, report the results from any model updating (i.e., model specification, model performance). No attempt was made to update any biomarker.	NAP
Discussion			
Limitations	18	Discuss any limitations of the study (such as nonrepresentative sample, few events per predictor, missing data).	9
Interpretation	19a	For validation, discuss the results with reference to performance in the development data, and any other validation data.	9
	19b	Give an overall interpretation of the results, considering objectives, limitations, results from similar studies, and other relevant evidence.	9
Implications	20	Discuss the potential clinical use of the model and implications for future research.	9
Other information			
Supplementary information	21	Provide information about the availability of supplementary resources, such as study protocol, Web calculator, and data sets. Comment: The data presented in this study are available on reasonable request from the corresponding author.	NAP
Funding	22	Give the source of funding and the role of the funders for the present study.	9

NAP = not applicable.

## Appendix B

**Table A2:** Predictors employed by the biomarkers.

	Biomarkers			
	FLI	LAP	HSI	ZJU
Triglycerides	✓	✓		✓
BMI	✓		✓	✓
GGT	✓			
Waist	✓	✓		
ALT:AST			✓	✓
T2DM			✓	
Sex			✓	
Glucose				✓

Abbreviations: BMI = body mass index; GGT = gamma-glutamyltransferase; ALT = alanine transaminase; AST = aspartate transaminase; T2DM = type 2 diabetes mellitus.

**Table A3:** Multivariable Prediction Models Employed by the Biomarkers.

Abbreviations and Units of Measurements	
altsalt	alanine transaminase (U/L)/aspartate transaminase (U/L)
bmi	body mass index (kg/m^2^)
exp	exponential operator
female	female gender (1 = female; 0 = male)
ggt	gamma-glutamyltransferase (U/L)
gmmol	glucose (mmol/L)
log_e_	natural logarithm
fli_lp	fatty liver index-linear predictor
t2dm	type 2 diabetes mellitus (1 = yes; 0 = no)
tg	triglycerides (mg/dL)
tgmmol	triglycerides (mmol/L)
wc	waist circumference (cm)
Fatty liver index (FLI)	
fli_lp	0.953*log_e_(tg) + 0.139*bmi + 0.718* log_e_(ggt) + 0.053*wc−15.745
FLI	[(exp(fli_lp)/(1 + exp(fli_lp)]*100
Lipid accumulation product (LAP)	
LAP	(wc−k)*tgmmol with k=65 if sex=male or k=58 if sex=female
Hepatic steatosis index (HIS)	
HSI	8*altast + bmi + 2*t2dm + 2*female
Zhejiang University index (ZJU)	
ZJU	bmi + gmmol + tgmmol + 3*altast +2*female
